# Artificial intelligence in orthopedics: fundamentals, current applications, and future perspectives

**DOI:** 10.1186/s40779-025-00633-z

**Published:** 2025-08-04

**Authors:** Jian Song, Guang-Chao Wang, Si-Cheng Wang, Chong-Ru He, Ying-Ze Zhang, Xiao Chen, Jia-Can Su

**Affiliations:** 1 Department of Orthopedics, Trauma Orthopedics Center, Institute of Musculoskeletal Injury and Translational Medicine of Organoids, Xinhua Hospital Affiliated to Shanghai Jiao Tong University School of Medicine, Shanghai, 200092 China; 2 Department of Orthopedics, Shanghai Zhongye Hospital, Shanghai, 200941 China; 3 Department of Orthopedics, Orthopedic Research Institution of Hebei Province, NHC Key Laboratory of Intelligent Orthopedic Equipment, The Third Hospital of Hebei Medical University, Shijiazhuang, 050051 China; 4 Institute of Translational Medicine, Organoid Research Center, National Center for Translational Medicine (Shanghai), Shanghai University Branch, Shanghai, 200444 China

**Keywords:** Artificial intelligence (AI), Orthopedics, Machine learning (ML), Deep learning (DL), Diagnostic, Therapeutics

## Abstract

Conventional diagnostic and therapeutic approaches in orthopedics are frequently time intensive and associated with elevated rates of diagnostic error, underscoring the urgent need for more efficient tools to improve the current situation. Recently, artificial intelligence (AI) has been increasingly integrated into orthopedic practice, providing data-driven approaches to support diagnostic and therapeutic processes. With the continuous advancement of AI technologies and their incorporation into routine orthopedic workflows, a comprehensive understanding of AI principles and their clinical applications has become increasingly essential. The review commences with a summary of the core concepts and historical evolution of AI, followed by an examination of machine learning and deep learning frameworks designed for orthopedic clinical and research applications. We then explore various AI-based applications in orthopedics, including image analysis, disease diagnosis, and treatment approaches such as surgical assistance, drug development, rehabilitation support, and personalized therapy. These applications are designed to help researchers and clinicians gain a deeper understanding of the current applications of AI in orthopedics. The review also highlights key challenges and limitations that affect the practical use of AI, such as data quality, model generalizability, and clinical validation. Finally, we discuss possible future directions for improving AI technologies and promoting their safe and effective integration into orthopedic care.

## Background

Artificial intelligence (AI) has experienced rapid progress in recent years, contributing to changes in multiple areas of medical practice through its ability to simulate human reasoning and aid in clinical judgments [1]. In 2016, preventable medical errors were reported to account for more than 250,000 deaths annually in the United States [2]. In health care, AI holds immense promise for enhancing the quality of care and minimizing such errors [3, 4]. Improvements in computing power and data access have led to rapid increases in the development of AI technologies and related medical devices, many of which have been approved by the United States Food and Drug Administration [5]. These innovations are gradually being integrated into various medical specialties. In particular, in orthopedics, a field long recognized for embracing technological progress, AI has begun to be applied to increase diagnostic accuracy and improve patient outcomes.

Although AI is increasingly used to support early diagnosis and precise treatment in orthopedics [6], the integration of AI into orthopedic practice presents unique challenges. Orthopedic conditions often involve complex biomechanical systems, patient-specific anatomical variations, and long-term recovery periods, complexities that demand robust and precise AI solutions. Challenges such as data scarcity, the complexity of unstructured clinical information, and integration issues with current workflows continue to restrict the practical use of AI in medicine. Moreover, ensuring the ethical use of patient data, regulatory compliance, and acceptance among healthcare providers further adds to the complexity.

Although challenges remain, various aspects of AI applications in orthopedics continue to show substantial potential. AI technologies enable more accurate diagnoses through advanced image recognition, optimize surgical interventions via predictive analytics, and personalize treatment plans using patient-specific data. Particularly in surgical applications, AI-based robotics and navigation systems are transforming the way orthopedic surgeries are planned and executed, improving precision and reducing recovery time [7, 8]. Collectively, these innovations align with the broader goals of precision medicine [9], offering tailored interventions to meet individual patient needs.

In recent years, interest in applying AI in orthopedic surgery has increased [6, 10–12]. This review examines current applications of AI in orthopedic diagnosis and treatment with a focus on its clinical benefits, technical limitations, and areas requiring further research and development (R&D). By identifying the current gaps in research and practice, we highlight key areas where AI has the potential to enhance orthopedic care and guide future efforts toward more effective integration of AI technologies.

## Definitions of AI

**Fig. 1:**
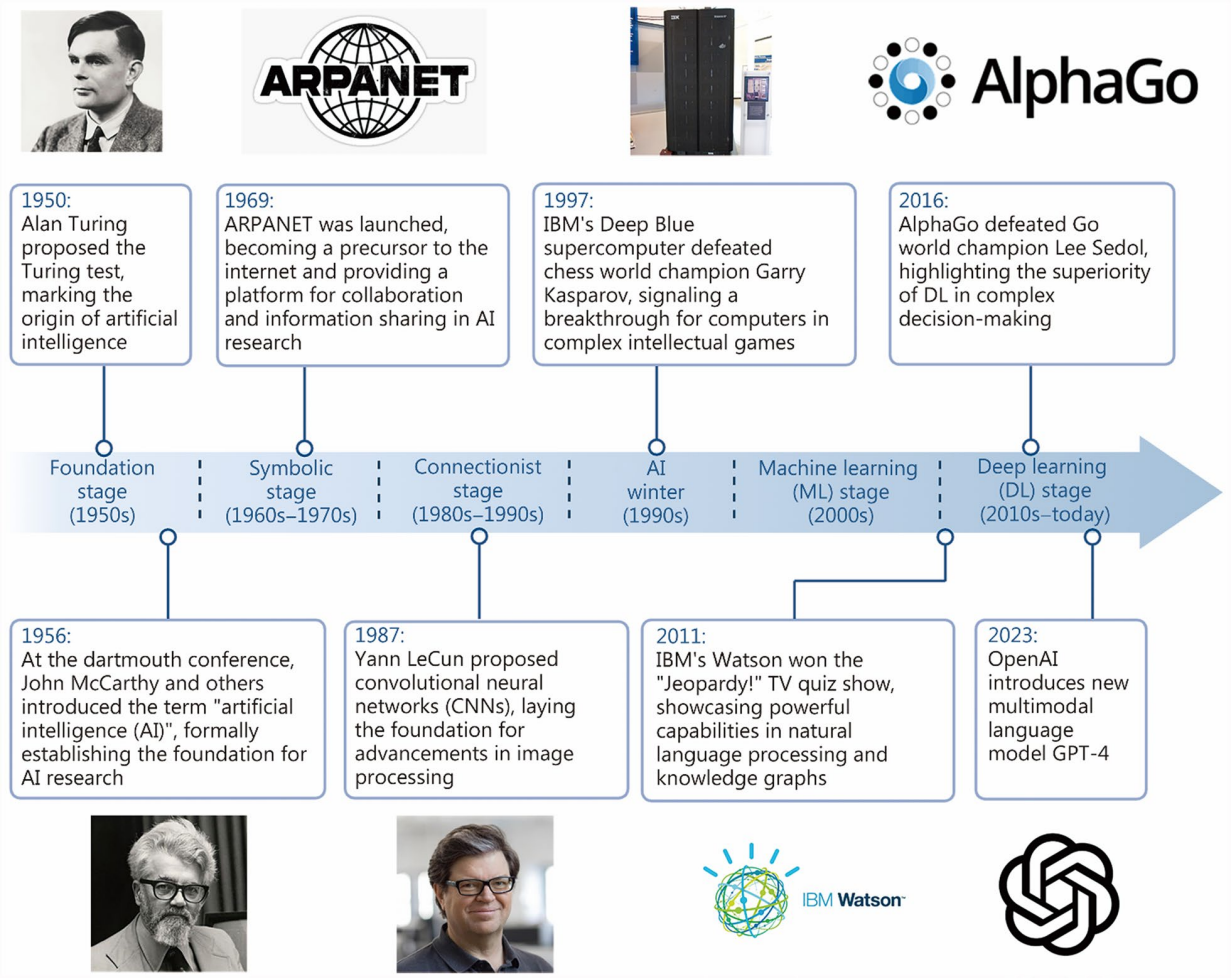
A brief history of AI development. GPT-4 generative pre-trained transformer 4

**Fig. 2:**
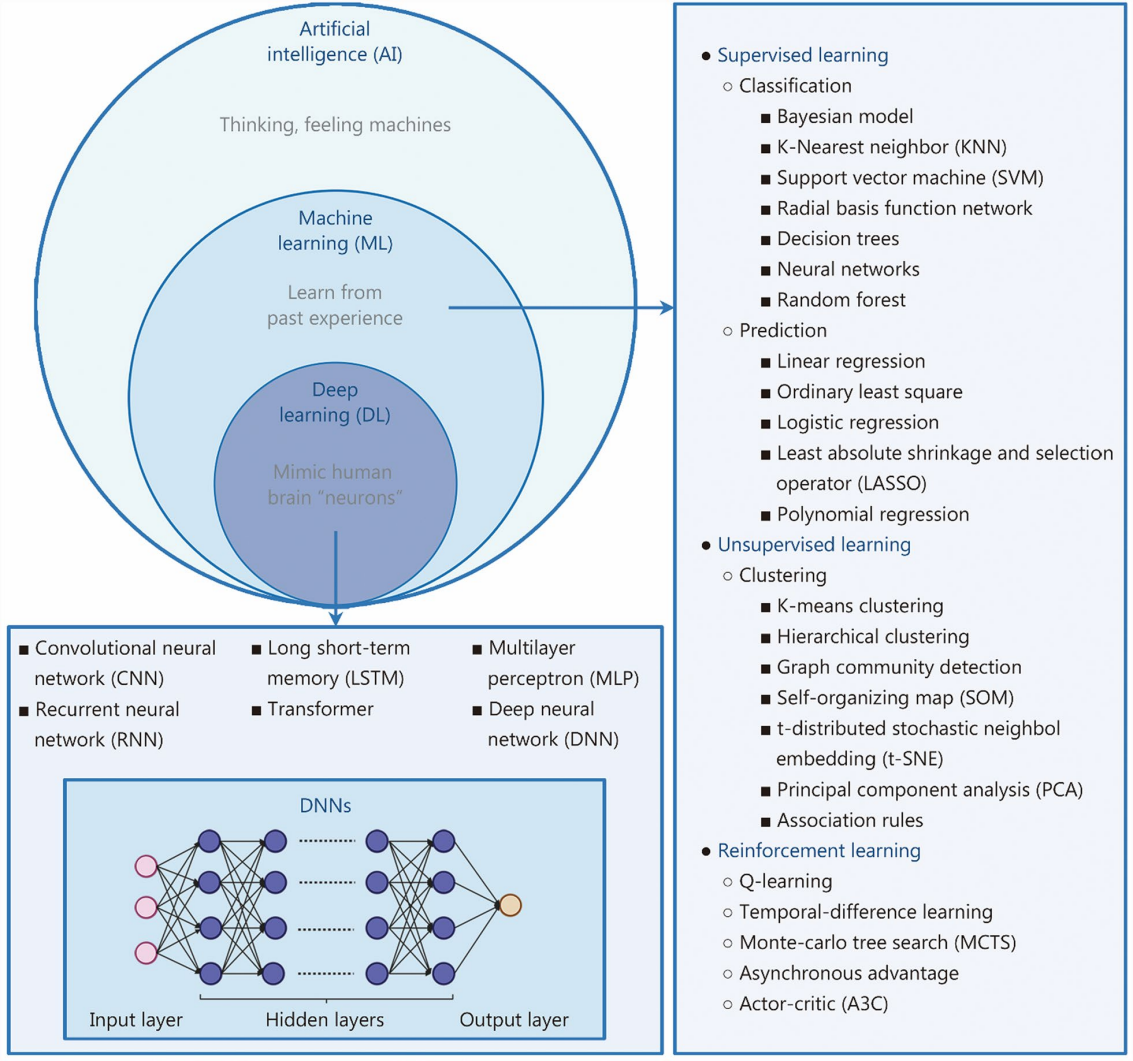
The relationship between AI, ML, and DL and commonly used algorithms as examples

The development of AI has been a gradual process [13] (Fig. 1). The foundation of AI traces back to McCarthy’s work in 1956, which marked the beginning of efforts to build machines that could emulate human thinking, imagined then as “thinking, feeling machines” [14–17] (Fig. 2). However, it was not until the 1980s that computerization and the automation of processes enabled AI to develop more widely [7]. Since the early 2000s, AI has rapidly advanced. By leveraging large datasets, deep learning (DL) has enabled machines to perform advanced tasks that require higher levels of pattern recognition and abstraction [18], with remarkable results in natural language processing, image recognition, and speech recognition. The past two decades have witnessed a steady increase in AI applications across various industries, including health care [19–21]. In orthopedics, the application of AI enhances diagnostic accuracy, enables personalized treatment planning, and supports predictive modeling, collectively improving patient outcomes and experiences.

### Machine learning (ML)

ML is a crucial subset of AI that empowers computer systems to learn from experience and acquire knowledge from datasets (Fig. 2). ML leverages algorithms to predict outcomes by identifying patterns in input data, and these predictions are compared with known outcomes to quantify and refine the accuracy of the algorithm [22]. ML manifests in two primary forms: supervised and unsupervised learning [22–24]. In supervised learning, algorithms are trained via labeled datasets [25]. The model learns to make predictions based on the provided examples, thus enabling autonomous decision-making. Supervised learning focuses on classification and prediction [26]. Conversely, unsupervised learning involves training algorithms on unlabeled data, allowing them to uncover hidden patterns or structures independently [27]. Unsupervised ML techniques are commonly employed for clustering similar data points or identifying relationships between variables within a dataset.

In health care, both supervised and unsupervised learning strategies have been extensively applied [26, 28]. Supervised ML facilitates the development of predictive models, enhancing capabilities in areas such as disease detection and therapeutic outcome forecasting [28, 29]. Conversely, unsupervised ML techniques enable the discovery of latent patterns within health care data, leading to insights such as disease subtyping and patient stratification [26, 28]. Recent studies have effectively employed supervised learning methods across various applications, including injury risk prediction among professional athletes, evaluation of knee osteoarthritis (OA) through kinematic analysis [30, 31], and prediction of clinically relevant outcomes [32–36]. In contrast, unsupervised learning has been used to identify distinct patient subgroups, aiding in risk stratification and personalized treatment. A recent study applied unsupervised clustering to knee ligament registry data and identified 5 patient subgroups with distinct anterior cruciate ligament (ACL) revision rates. The classification was based on age, graft type, and preoperative function scores [37].

### DL

DL is a multilayer representation learning method that transforms raw data into abstract features through structures that mimic human brain neurons, enabling automatic pattern recognition and classification (Fig. 2). DL utilizes artificial neural networks to extract complex features from large, high-dimensional data [18]. These networks consist of millions of interconnected neurons arranged in layers. Each neuron receives input from the previous layer and passes processed information to the next. During training, data with known outcomes are fed into the input layer and processed through hidden layers before a prediction at the output layer is produced [38].

There are several foundational types of neural networks, each suited to different data types and tasks. The multilayer perceptron forms the basis for many DL models through its fully connected structure [20]. Recurrent neural networks are designed to handle sequential data by processing one element at a time while retaining information from previous inputs through hidden states [39]. In contrast, convolutional neural networks (CNNs) are particularly effective for image analysis because of their ability to capture local spatial features [38]. These models are often used independently or combined as components in more advanced DL architectures. These neural network architectures have enabled significant advances in various fields, including orthopedics, where DL has been widely applied to tasks such as image analysis [40, 41], diagnosis [42–44], surgery [45], drug development [46–48], and predictive analytics [49].

## Algorithm development for orthopedic image analysis

Recent developments in AI have facilitated progress in orthopedic imaging, particularly in the automated segmentation and localization of anatomical structures (Table 1) [40, 41, 50–58]. AI offers practical tools for streamlining these complex tasks and supports the application of ML and DL techniques in the diagnosis and treatment of orthopedic conditions.

**Table 1:** Application of AI in the analysis of orthopedic images

Algorithm	Application	References
ML model	Measuring and segmenting articular cartilage thickness in healthy knees	[58]
DL model	Segmenting cartilage and meniscus in knee MR images	[41]
DCNN	Segmenting pelvic muscles, fat, and bone	[40]
U-Net and SegResNet architectures	Segmenting fumer	[53]
ML model	Identifying and labeling vertebrae and intervertebral discs on MR images	[57]
Algorithms based on regression forests and probabilistic graphical models	Locating and identifying vertebrae in CT scans	[54]
ML model	Segmenting the spine and identifying the pedicles	[50]
DL model	Segmenting lumbosacral nerves	[52]
CNN	Identifying and labeling intervertebral discs and predicting multiple pathology grades	[55]
ResNet-based neural network	Identifying and classifying various nested fracture categories	[56]
DL model	Classifying foot types	[51]

*ML* machine learning, *DL* deep learning, *MR* magnetic resonance, *DCNN* deep convolutional neural networks, *CNN* convolutional neural networks, *CT* computed tomography

### Progress in segmentation and classification algorithms

Orthopedic image analysis has benefited from the refinement of segmentation algorithms that enable the delineation of structures such as cartilage, bone, muscle, and neural elements. These advances are grounded in both traditional ML models and modern DL architectures.

For articular cartilage segmentation, Shah et al. [58] utilized a validated ML model to quantify cartilage thickness from 3910 magnetic resonance imaging (MRI) datasets of healthy knees. This approach enabled differentiation between tissue layers, supporting longitudinal observations of joint degeneration. However, ML-based models often lack adaptability, and their dependence on manual feature extraction limits scalability. In contrast, Norman et al. [41] implemented a U-Net-based DL model for segmenting cartilage and menisci. The model achieved Dice coefficients between 0.770 and 0.878 for cartilage and 0.753 and 0.809 for menisci, with segmentation times averaging 5 s per scan. It also showed a strong correlation with manual relaxometry and morphology measurements, demonstrating its potential for reliable OA assessment in clinical workflows (Table 1).

For bone structure segmentation, Ghidotti et al. [53] compared the U-Net and SegResNet architectures and achieved high spatial accuracy. SegResNet, with its enhanced skip connections, demonstrated superior adaptability under specific training conditions, underscoring its potential for precise three-dimensional (3D) anatomical modeling in orthopedic applications. Similarly, in pelvic segmentation, Hemke et al. [40] developed a deep convolutional neural network (DCNN) model capable of segmenting pelvic computed tomography (CT) images into multiple tissue classes with high accuracy, completing segmentation of each image in under 0.1 s on a graphics processing unit. These results highlight the robustness and efficiency of the model, reinforcing the value of DL in comprehensive body composition analysis (Table 1).

Spinal segmentation, particularly under pathological and incomplete conditions, remains challenging. Early approaches, such as Oktay and Akgul’s Markov-chain-like graphical model with handcrafted pyramidal histogram of oriented gradient (PHOG) and image projection descriptor features, addressed disc and vertebra localization in abnormal magnetic resonance datasets [57]. Building on this, Glocker et al. [54] and Burström et al. [50] introduced automated methods for CT and cone-beam computed tomography (CBCT) that facilitated pedicle screw navigation. To overcome the limitations of handcrafted features, Jakubicek et al. [59] proposed an innovative multistage DL framework integrating 3 CNNs with novel spatial filtering and global optimization methods, achieving a mean intervertebral disc localization error of 4.4 mm and 87.1% vertebra labeling accuracy on challenging pathological and incomplete 3D CT spine scans. Complementing these efforts, Fan et al. [52] demonstrated high-accuracy segmentation of lumbosacral nerves and bone, with performance statistically comparable to that of manual segmentation, reinforcing the clinical potential of DL-based models. Across studies, multistage CNN frameworks exhibit better resilience than other frameworks in handling abnormal spine morphology (Table 1).

Beyond anatomical segmentation and localization, AI has also been increasingly applied to automated detection and classification tasks, expanding its utility in clinical decision-making. Jamaludin et al. [55] developed a CNN-based system that achieved 95.6% accuracy in disc detection and labeling from MR images, and its pathological grading predictions closely matched radiologists’ evaluations, offering consistent voxel-level “evidence hotspots” to support clinical interpretation. Similarly, Lind et al. [56] created a ResNet-based neural network that classified 49 nested fracture categories around the knee, achieving weighted mean area under the curves (AUCs) of 0.87 for proximal tibia fractures, 0.89 for patella fractures, and 0.89 for distal femur fractures, with nearly 75% of the AUC estimates exceeding 0.8. These results demonstrate the model’s capacity for detailed and reliable fracture classification based on radiographic data. Expanding into multimodal data, Chae et al. [51] combined imaging and digital foot pressure data to classify foot types, demonstrating the adaptability of AI beyond conventional imaging tasks (Table 1).

These studies illustrate the growing potential and complexity of AI in orthopedic diagnostics, particularly when diverse data types are integrated and atypical pathologies are addressed. Despite these advancements, certain limitations and challenges remain to be addressed to ensure consistent and accurate performance across diverse clinical scenarios.

### Technical limitations in image analysis

Despite recent advancements in image segmentation and its increasing accuracy and efficiency in identifying anatomical structures and assisting in clinical decisions, several persistent limitations remain across current studies. These limitations are related to data dependency, generalizability, model design, and clinical applicability. To begin with, a number of investigations have faced difficulties related to annotation accuracy [41, 56]. Certain works have used manual segmentation as the reference standard, which is inherently subjective and lacks consistency across annotators, thus limiting its validity as a ground truth [41]. Reliance solely on radiology reports for annotation, without corroborating evidence from CT, MRI, or surgical findings, raises the risk of misclassification, particularly when dealing with complex anatomical structures or fine-grained subclassifications [56]. Furthermore, the representativeness and pathological diversity of training datasets are often insufficient. Studies based on data from a single institution may be influenced by specific imaging protocols, patient populations, and equipment settings, limiting their broader applicability [40, 56]. Additionally, certain works have limited the scope of segmentation to single anatomical planes or specific structures, such as assessing muscle quality using only a standard axial hip slice [40] or segmenting exclusively the L5/S1 spinal level, which prevents the integration of multiview or 3D information essential for comprehensive analysis [41, 55]. For example, Burström et al. [50] trained a model on cadaveric images without overt pathology, which, although effective for general anatomical segmentation, may underperform in cases of disease such as spinal deformities or degenerative lesions.

Equally important is the lack of standardization in evaluation metrics, which limits comparability across studies. Some studies have prioritized classification accuracy but neglected consistency metrics such as the kappa coefficient, potentially obscuring performance differences across categories and limiting transparency regarding clinical reliability [55]. Moreover, the clinical utility of many existing models has only been preliminarily evaluated and still needs confirmation through future large-scale, prospective validation. For example, Lind et al. [56] proposed DL-assisted plane localization for planning epidural steroid injections, but its feasibility remains to be established through extensive trials. Similarly, Hemke et al. [40] acknowledged the need to validate the prognostic utility of muscle quality assessment models in patients with cancer or chronic diseases.

## AI-powered innovations in orthopedic diagnostics

Diagnostic errors occur across all phases of surgical care and specialties, with clinical decision-making and communication breakdown as leading contributors, resulting in at least moderate harm in over half of cases and death in 1 in 7 patients [49, 60]. The accelerated advancement of AI technologies, coupled with the vast expansion of medical data [61], has driven increasing integration of AI into diagnostic research and clinical applications [62].

### Facilitating precise and efficient fracture diagnosis

Fracture diagnosis remains a cornerstone of orthopedic practice, yet conventional radiographic interpretation is prone to misdiagnosis due to interobserver variability, complex anatomical structures, and heavy clinical workloads [63, 64]. The global age-standardized incidence rate of fractures in 2019 was estimated at 2296.2 cases per 100,000 people [65], and diagnostic errors can lead to delayed treatment and poor recovery of function [44, 66]. The incorporation of AI into fracture diagnosis represents a promising advancement aimed at enhancing diagnostic precision, promoting consistency in interpretation, and streamlining clinical processes [67, 68]. Many studies have shown that AI-based models perform strongly in detecting acute fractures, achieving accuracies comparable to those of expert radiologists (Table 2) [42–44, 69–78].

**Table 2:** Application of AI in the diagnosis of fractures

Fracture site	Algorithm	References
Fractures of upper limb bones	CNN, DNN	[42–44, 71]
Rib fractures	DL model	[75, 78]
Thoracolumbar fractures	ML model	[70, 74]
Hip fractures	CNN	[69, 77]
Scaphoid fractures	DL model	[73]
Calcaneal fractures	CNN	[76]
Fractures across the musculoskeletal system	DL model	[72]

*CNN* convolutional neural network, *DNN* deep neural network, *DL* deep learning, *ML* machine learning

AI models trained on X-ray and CT datasets have successfully identified fractures of varying sizes and complexities. In a study by Chung et al. [42], a DCNN demonstrated proficiency in detecting proximal humerus fractures. Similarly, Lindsey et al. [44] trained a deep neural network (DNN) capable of detecting and localizing wrist fractures in radiographs with diagnostic accuracy comparable to that of advanced subspecialty plastic surgeons. This study also demonstrated that emergency medicine clinicians aided by the trained model achieved a sensitivity of 92.5% and a specificity of 94.1%. Another study by Kim et al. [43] demonstrated that transfer learning, where a CNN pretrained on nonmedical images was fine-tuned on medical datasets, could produce highly accurate fracture diagnoses, even with limited labeled radiographs. Furthermore, Guan et al. [71] proposed a novel DL approach for the X-ray-based detection of arm fractures and achieved a state-of-the-art average accuracy of 62.04% for arm fracture detection, even for a musculoskeletal radiograph (MURA) dataset with images of low quality. In contrast to studies that reported higher sensitivity and specificity based on high-resolution data or more distinct anatomical targets, the study by Guan et al. [71] focused on overcoming real-world imaging constraints. Following the application of AI models in upper limb fracture detection, AI models have also been applied for detecting rib fractures, an especially challenging task owing to the high miss rate of up to 50% on conventional chest radiographs, which are limited by overlapping structures, reader variability, and image quality [79, 80]. Niiya et al. [75] introduced a system that achieved a sensitivity of 93.5%, significantly improving fracture detection efficiency in trauma settings. Similarly, Yao et al. [78] constructed a DL-based rib fracture detection system using a 3-step algorithm.

Moreover, AI has shown promise in the diagnosis of thoracolumbar vertebral fractures, which are frequently missed or misdiagnosed on plain radiographs because of their suboptimal sensitivity, potentially leading to delayed treatment and a worse prognosis [81, 82]. Furthermore, vertebral compression fractures, particularly those involving significant anterior height restoration and low bone mineral density, not only increase the risk of subsequent adjacent vertebral fractures but also significantly increase the risk of future hip fractures [83, 84]. Therefore, early diagnosis of thoracolumbar compression fractures is crucial. Burns et al. [70] utilized an automated ML computer system to detect spinal fractures with 95.7% sensitivity. Additionally, Li et al. [74] developed an AI-based lumbar vertebral fracture detection system that demonstrated 92% accuracy, 91% sensitivity, and 94% specificity.

DL algorithms have been widely applied to multisite fracture detection. Studies have shown that CNN and ResNet models achieve high accuracy in diagnosing fractures of the femoral neck, hip, scaphoid, and calcaneus [63, 73, 76, 77]. Jones et al. [72] reported an AUC of 0.974 for a DL model targeting 16 anatomical fracture sites. The sensitivity and specificity were 95.2% and 81.3%, respectively. In a secondary analysis of radiographs with complete expert agreement, the AUC further increased to 0.993, which closely approximates the diagnostic accuracy of experienced radiologists and orthopedic surgeons.

Nonetheless, AI-based fracture diagnosis still faces several unresolved challenges [85]. First, real-world, multicenter validation is essential to ensure that AI systems perform reliably in clinical settings, as unexpected behaviors may not surface during standard evaluations [86]. Second, cost concerns and lack of transparency remain key barriers to AI adoption in radiology. Real-world improvements in clinical efficiency and diagnostic quality are essential to justify investment and promote broader implementation [87]. Furthermore, current AI systems struggle to detect subtle or atypical fractures, such as stress fractures or those in pathologic bone, owing to limited training exposure. Moreover, most AI tools are narrowly designed for specific tasks such as image classification and fall short of replicating the full scope of radiologists’ responsibilities, which include clinical correlation and complex decision-making [85].

### Assisting in the early diagnosis of developmental dysplasia of the hip (DDH)

DDH, the most common pediatric musculoskeletal disorder, can lead to severe disability if not diagnosed and treated early; timely and accurate detection is therefore critical for preserving hip joint function and preventing long-term complications [88, 89]. Traditional diagnostic methods, such as hip ultrasound for infants and pelvic radiographs for older children, rely heavily on clinician expertise, making them time-consuming, labor-intensive, and prone to variability [90]. The application of AI in imaging-based DDH screening contributes to greater diagnostic accuracy and improves workflow efficiency. Huang et al. [91] introduced DDHnet, a fully automated AI system that measures 4 key hip parameters (α-angle, β-angle, femoral head coverage, and pubofemoral distance) from ultrasound images. DDHnet reached a diagnostic accuracy of 98.64%, with 100.00% specificity and 90.56% sensitivity. These results demonstrate the ability of the system to deliver precise and consistent measurements while significantly reducing the time required for analysis. Xu et al. [92] further developed an AI model that measures additional parameters such as the acetabular index, center–edge angle, Tönnis grade, and International Hip Dysplasia Institute (IHDI) grade, demonstrating diagnostic performance comparable to that of orthopedic specialists but with a significantly reduced processing time, the model required only 1.21 s per case, compared with 150.36 to 200.71 s for surgeons of varying experience levels (*P* < 0.001).

AI also has the potential to increase access to DDH screening in diverse clinical settings. Jaremko et al. [93] published a study showing that MEDO-Hip (MEDO.ai, Edmonton, Canada, 2021), an AI-based ultrasound evaluation software program, enables primary care workers with simple training to screen infants for DDH. This study demonstrated that the AI-based portable ultrasound screening can achieve follow-up and case detection rates similar to those of specialized ultrasound screening. MEDO-Hip facilitates population-level DDH screening, which is expected to reduce screening costs and enable widespread implementation.

AI performs well in objective measurements but struggles with subjective assessments [92]. More training data is needed to improve its ability in complex cases. Image quality remains a critical factor, as poor-quality scans can lead to inaccurate results. AI systems are highly dependent on the presence of all key anatomical landmarks. Moreover, most studies are on a small scale and rely on specific software‒hardware combinations, limiting their generalizability to other clinical settings and devices [93–95].

### Enhancing soft tissue injury diagnosis

Meniscal tears resulting from trauma and degeneration frequently cause knee pain in patients [96]. Accurate diagnosis and appropriate treatment are essential for improving patients’ quality of life [97]. The CNN model proposed by Bien et al. [98] for ACL and meniscal tear detection achieved AUCs of 0.965 and 0.847, respectively, on the internal validation dataset. However, the model was evaluated with only one MR image, limiting its clinical application. To address these limitations, Fritz et al. [99] conducted a clinical validation study of a fully automated DCNN for detecting surgically confirmed meniscal tears. Compared with surgical findings and assessments by musculoskeletal radiologists, the DCNN model demonstrated fully automated detection of meniscal tears, achieving similar specificity but reduced sensitivity. Pedoia et al. [100] used a CNN for the diagnosis of meniscal injuries. They achieved 80% accuracy and reported a tendency for the misdiagnosis of posterior horn meniscal tears.

Among sport-related musculoskeletal injuries, ACL tears are common but challenging to diagnose [101, 102]. Researchers have developed various diagnostic models to aid in clinical analysis. The groundbreaking study by Štajduhar et al. employed a semiautomated method using MRI data to detect ACL injuries [103]. The research utilized histograms of oriented gradients (HOGs) and global image statistics methods for feature extraction, combined with support vector machines (SVMs) and random forests, and achieved an AUC of 0.943 in detecting ACL injuries, including mild and complete ruptures. Richardson et al. [104] demonstrated that CNNs can effectively replace human readers in MRI protocol optimization, particularly in detecting ACL tears, where fat-saturated sequences outperform non-fat-saturated sequences in terms of sensitivity. To enhance generalizability, Tran et al. [105] trained the algorithm using a multicenter dataset consisting of 19,765 knee MRI scans from 12 centers, incorporating diverse scanner types (1/1.5/3 T) and imaging protocols, demonstrating high performance across external datasets.

In addition to MR image-based diagnosis, many studies have established diagnostic models based on other criteria. Liu et al. [106] utilized arthroscopy as a reference standard, Zeng et al. [107] explored gait analysis to train their model, and Li et al. [108] based their approach on plantar pressure monitoring. These alternative methods expand the potential applications of AI beyond traditional imaging and contribute to the development of comprehensive, multimodal diagnostic strategies.

Although some models are trained on large and diverse datasets, their performance is often evaluated on the basis of data from single institutions, raising concerns about generalizability. Notably, improved diagnostic accuracy following retraining on external datasets suggests that model adaptation to local imaging protocols and population characteristics may be necessary [98]. These limitations underscore the need for larger, multi-institutional studies with standardized reference standards and broader clinical validation to ensure reliable real-world performance of AI models in musculoskeletal imaging.

### Improving OA diagnosis and grading

In 2020, OA accounted for 4.3% of the number of years lived with disability worldwide, reflecting a 9.5% increase since 1990 and underscoring its growing impact on public health worldwide [109]. Therefore, it is particularly important to automate the detection of OA via AI. Conrozier et al. [110] introduced a method to diagnose early OA by measuring the joint gap width. The disadvantage of this method is that it requires frequent interventions by the observer to select the region of interest and adjust the bone edge detection. Unlike manual methods, which heavily depend on observer input to select regions of interest and adjust bone edge detection, this AI-driven algorithm provides an automated and objective measurement of joint space width, reducing user dependency and improving reproducibility [111]. Üreten et al. [112] developed a CNN-based computer-aided diagnostic method using transfer learning for hip OA detection on plain pelvic radiographs. Their model achieved 90.2% accuracy, 97.6% sensitivity, and 83.0% specificity, demonstrating the potential to assist clinicians with objective interpretation and reduce the need for advanced imaging.

Recent developments have extended AI applications to OA severity grading. To automate the evaluation of knee OA severity, Tiulpin et al. [113] introduced a DL framework utilizing CNN aligned with the Kellgren-Lawrence classification system. Their approach yielded a multiclass accuracy of 66.71%, a Cohen’s kappa (quadratic) value of 0.83, and an AUC of 0.93, indicating substantial concordance with specialist-provided labels. Similarly, another automatic Kellgren-Lawrence grading model using the DenseNet neural network architecture achieved sensitivities from 68.9 to 86.0% and specificities ranging from 83.8 to 99.1% across different severity levels, indicating strong classwise discriminatory ability [114]. A DenseNet-based model was proposed by Pedoia et al. [115] for early-stage knee OA detection, employing T2-weighted MRI imaging to facilitate diagnosis before conventional radiographic signs are evident. When patient demographic data were integrated, their model exhibited 76.99% sensitivity and 77.94% specificity across a vast patient cohort from the OA Initiative baseline dataset. These studies highlight the utility of ML techniques, particularly DL-based approaches, in automating the detection and severity grading of OA from medical imaging data. These advancements hold promise for facilitating early diagnosis, personalized treatment strategies, and improved clinical management of patients with OA.

## AI-based advances in orthopedic treatment

### Enhancing orthopedic surgery quality

The application of AI in orthopedic surgery has transformed the quality of care by improving precision, enhancing surgical planning, and streamlining intraoperative workflows [116–120]. Robotic platforms, navigation technologies, and AI-powered imaging analysis have become integral to optimize patient-specific interventions (Table 3) [45, 121–128].

**Table 3:** Application of AI in orthopedic surgery

Surgery	Algorithm/robot	Application	References
THA	3D hip surgery simulation system	3D simulation for acetabular reconstruction in dysplastic hips	[121]
THA	CNN	Identifying the design of a failed THA implant within seconds before surgery	[45]
TKA	Robotic arm	Providing good positioning, improving prognosis, and reducing bone and soft tissue damage	[125]
TKA	Robotic arm	Improving the mechanical alignment, range of motion, and functional outcomes	[126]
RSA	Navigation technology	Improving the accuracy of glenoid component placement	[124]
SA	Robotic platforms	Real-time manufacturing of patient-specific instruments	[122]
Ankle joint reset	Image-guided robotic assistant	Assisting ankle reduction with the contralateral ankle as a reference	[123]
Tendon graft	Robotic biomechanical testing system	Simulating 3D kinematics and contact pressure	[127]
ACL reconstruction	Robot-assisted systems	Providing real-time feedback and automatic adjustments during surgery	[128]

*THA* total hip arthroplasty, *3D* three-dimensional, *CNN* convolutional neural network, *TKA* total knee arthroplasty, *RSA* reverse shoulder arthroplasty, *SA* shoulder arthroplasty, *ACL* anterior cruciate ligament

#### Lower limb arthroplasty

AI has significantly advanced total hip arthroplasty (THA) and total knee arthroplasty (TKA) by improving preoperative planning, implant selection, and surgical precision. Chen et al. [121] investigated the relationship between the typology of DDH cases and the internal and external diameters of the socket via a 3D hip surgery simulation system, which demonstrated superior accuracy in prosthesis implantation compared with conventional two-dimensional (2D) templates. This transition from 2D to AI-enhanced 3D simulations allows for patient-specific solutions in complex developmental DDH cases, reducing errors in prosthesis placement.

For THA, AI-based tools have optimized implant recognition and alignment. Borjali et al. [45] developed a CNN-based algorithm which was evaluated on an independent test set of 252 anteroposterior hip radiographs and achieved 100% accuracy in identifying 3 common THR implant designs. These controlled test conditions demonstrate the model’s strong performance of the model, although further validation in larger clinical datasets is needed. Robot-assisted techniques have led to notable improvements in TKA, particularly in achieving more accurate mechanical alignment, minimizing intraoperative trauma, and enhancing early postoperative recovery. Systems such as Zimmer Biomet’s Robot of Stereotactic Assistant (ROSA)® and Stryker’s Mako® systems provide real-time haptic feedback during bone cutting and soft tissue balancing, enhancing intraoperative decision-making. Kayani et al. [125] confirmed that robotic arm-assisted TKA leads to significantly reduced surgical trauma compared with conventional jig-based TKA. Specifically, patients in the robotic group experienced lower postoperative pain and analgesia requirements and smaller reductions in postoperative hemoglobin levels, suggesting less intraoperative blood loss and faster functional recovery, including a shorter time to straight leg raise and earlier discharge. Rossi et al. [126] confirmed that robotic-assisted TKA improved mechanical alignment and range of motion in patients with severe varus and valgus deformities, achieving a 100% short-term survival rate with no major complications at a minimum follow-up of 6 months. Collectively, these advancements highlight the critical roles of AI and robotics in THA and TKA. AI tools aid in preoperative planning, ensuring accurate implant selection and alignment, whereas robotic systems provide real-time intraoperative guidance, improving precision and minimizing complications.

However, despite the significant role of AI and robotics in joint replacement, current research still has limitations. Limited sample size and few complex cases reduce model generalizability and recognition accuracy [45, 121]. Multicenter and multioperator involvement introduce variability, affecting measurement standardization [121, 125]. Lack of blinding, absence of correlation between short- and long-term outcomes, and inconsistent anesthesia and rehabilitation protocols restrict generalizability [125]. Future research should expand samples, standardize procedures, incorporate multimodal imaging, and strengthen long-term follow-up to increase the clinical value of these technologies.

#### Shoulder arthroplasty

The integration of navigation systems and robotic platforms in reverse shoulder arthroplasty has improved the accuracy of glenoid component placement, reducing manual errors such as improper inclination and off-center positioning. Giorgini et al. [124] demonstrated that computer-assisted navigation significantly improves the accuracy of glenoid component placement, reducing the number of errors associated with manual techniques. However, as Twomey-Kozak et al. [129] noted, despite these promising findings, the application of navigation systems in shoulder arthroplasty remains in its early stages, especially compared with their established use in hip and knee replacements. Darwood et al. [122] emphasized the benefits of robotic platforms for the real-time manufacturing of patient-specific instruments, reporting that the system achieved version and inclination angle accuracies of 1.9° [standard deviation (SD) 1.3] and 1.2° (SD 0.7), respectively, along with a positional accuracy of 1.1 mm (SD 0.7) relative to the preoperative plan.

Although these innovations hold promise, shoulder arthroplasty faces unique challenges that impact the effectiveness of AI-based navigation. Giorgini et al. [124] highlighted that humeral component navigation is still underdeveloped, limiting the ability to achieve optimal joint stability. Surgeons must manually align the humeral implant, which introduces variability in surgical precision. Additionally, the initial adoption of AI-based navigation systems increases the operative time, as surgeons must adapt to new workflows. Future research should aim to expand navigation capabilities to both the humeral and glenoid components, optimizing joint biomechanics and implant longevity. The integration of augmented reality and virtual reality for intraoperative guidance could enhance real-time surgical precision and efficiency [129].

#### Fracture reduction

Achieving excellent reduction is crucial for fracture healing. Ankle trauma is highly prevalent, with an estimated 2 million acute sprains occurring annually in the United States [130]. Due to the complexity of the anatomy, even minor injuries can lead to serious consequences [131], and up to 70% of patients may experience residual physical disability. Improper reduction of the joint during surgery can result in an insufficient contact area or excessive contact force, thereby increasing the risk of postoperative complications [132, 133]. AI-based fracture reduction has been developed to minimize intraoperative fluoroscopy exposure while improving reduction accuracy. Gebremeskel et al. [123] proposed an image-guided robotic system for ankle fracture reduction, utilizing the contralateral ankle as a reference to determine optimal manipulative forces and displacement parameters. This study quantified the manipulative forces and their displacement necessary to reduce the ankle symphysis accurately, marking a crucial first step in defining the design requirements for robotic assistance in terms of force and displacement. Robotic-assisted fracture reduction has notable limitations. The effectiveness of AI-based force prediction models is dependent on the variability of individual patient anatomy, and real-time adaptation remains a challenge. Additionally, current robotic reduction techniques lack intraoperative feedback mechanisms, which may lead to unintended overcorrection or soft tissue complications. Future efforts should focus on real-time AI adjustments using intraoperative fluoroscopic or CT-based feedback systems to ensure optimal reduction without excessive manual interventions.

#### Ligament reconstruction

AI has improved ligament reconstruction procedures by enhancing surgical planning and intraoperative precision. Sakakibara et al. [127] introduced a robotic biomechanical testing system for simulating joint kinematics and tendon graft placement, suggesting reconstruction at 30° of plantar flexion for optimal outcomes. Additionally, Yang et al. [128] utilized AI-based 3D CT and MRI analysis for personalized ACL reconstruction planning, integrating robotic assistance for real-time surgical adjustments. Their study demonstrated that robotic guidance improved bone tunnel drilling accuracy, maintaining deviations within 1.5 mm.

### Accelerating drug development

New drug development is a long and complex process characterized by high R&D costs and significant uncertainty [134]. In recent years, traditional methods of new drug R&D have become increasingly difficult, with increasing investments and extended development times. The field is currently at a bottleneck stage, necessitating new technologies to achieve cost reduction and increased efficiency. The rapid advancement of AI technology has introduced new opportunities for high-quality developments in the biopharmaceutical industry [135]. As early as the 1980s, Merck began to design drugs using computer-aided design [136]. With the progression of computer technology, AI has gradually predominated, becoming increasingly involved in the drug development process [137]. This approach ensures the quality of analysis while significantly reducing drug R&D costs, shortening development time, and improving overall efficiency, thereby putting new drug development on a fast and efficient path [138, 139]. AI has found applications in predicting protein structure and functional properties [46–48, 140–142], forecasting drug‒protein interactions [143, 144], and facilitating high-throughput drug screening [145].

#### Predicting protein 3D structures, properties, and functions

Understanding the 3D structures, properties, and functions of proteins is a critical step in drug discovery and development. An important example of such efforts is the study by Jumper et al. [46], who developed AlphaFold, a neural network model that provides a computational method for predicting protein structures with atomic accuracy, even in the absence of a known analogous structure. The results showed that, in most cases, its accuracy is comparable to that of the experimental structures and greatly outperforms that of the other methods.

In 2023, Yuan et al. [142] proposed a DL framework that combines bidirectional temporal convolutional networks, bidirectional long short-term memory, and a multiscale bidirectional temporal convolutional network for secondary protein structure prediction, achieving superior performance over existing methods on the Critical Assessment of protein Structure Prediction 10 – 14 (CASP10 – 14) and CullPDB 513 (CB513) benchmark datasets. Wang et al. [48] developed a DL framework, a language model with a geometric vector perceptron, consisting of a protein language model and a graph neural network, which can make predictions about protein properties by utilizing one-dimensional amino acid sequences and 3D structural information of proteins. Gligorijević et al. [140] introduced deep functional residue identification (DeepFRI), a graphical convolutional network that outperforms current major methods and sequence-based CNNs. DeepFRI uses sequence features extracted from protein language models and protein structures to predict protein functions. AI technology has great potential for accurately predicting protein structures and functions, which will lead to unprecedented technological innovations for new drug development.

#### Predicting drug-protein interactions

Identifying the target proteins of a drug and predicting the drug-target protein interactions play extremely important roles in the drug development process. By predicting drug-receptor or -protein interactions, researchers can better understand the efficacy of a drug and design it most effectively. For example, Offensperger et al. [143] discovered hundreds of interactions between fragments and proteins through a large-scale chemical proteomics survey. They reported that the data generated are suitable for ML-based models, which, when the chemical structure is used as input, can predict how the chemical interacts with the native proteome in intact cells. Investigating protein–ligand interactions, Wang et al. [144] trained an SVM model on 15,000 ligand–protein interactions involving 626 proteins and 10,000 active compounds. This model successfully identified 9 new compounds and their interactions with 4 key targets (G protein-coupled receptor 4, sirtuin 1, p38 mitogen-activated protein kinase, and glycogen synthase kinase 3 beta). Predicting drug-protein interactions will accelerate the drug development process by reducing the time required for target screening.

#### High-throughput drug screening

Although AI has not yet been widely applied to screen drug targets for orthopedic diseases, its notable success in other disease areas has driven a growing number of companies and large pharmaceutical firms to invest in AI-based drug discovery efforts [135]. The PandaOmics platform exemplifies how multiple AI engines can accelerate drug development by identifying promising targets [145]. In the case of INS018_055 [145], PandaOmics integrated multiomics data, literature trends, and biological network analysis to identify TNIK as a novel therapeutic target within just 18 months.

Although AI technology provides unprecedented opportunities for the discovery and development of novel orthopedic drugs by significantly improving the efficiency of drug screening and optimization, several challenges remain. First, in terms of data, there is a notable scarcity of high-quality, consistent, and accessible datasets [137, 146]. Unlike in fields such as image recognition, drug discovery suffers from limited annotated experimental data due to the inherent complexity of biological systems and variability in experimental conditions, leading to inconsistent and unreliable results. Second, challenges with molecular representation persist. Model performance is highly sensitive to the type and quality of molecular input used. Most current representations fail to account for essential aspects such as stereochemistry, conformational flexibility, the molecular surface, and compactness, all of which are crucial to molecular function [146]. Moreover, selecting an appropriate representation often involves a trade-off between simplicity and expressiveness, complicating the goal of model interpretability [137]. To fully realize the potential of AI, a collaborative effort is necessary to improve data quality, increase model transparency, and foster interdisciplinary collaboration.

### Contributing to intelligent rehabilitation

Surgery is not the endpoint of orthopedic disease treatment. Postoperative complications such as joint stiffness and ossification can severely affect patient prognosis, making rehabilitation a crucial part of orthopedic postoperative care. With advancements in AI R&D, rehabilitation robots and other AI-based technologies are playing a significant role in assisting in rehabilitative treatment.

Recent advancements in intelligent rehabilitation systems have demonstrated the potential of AI to support complex motor function recovery. Averta et al. [147] designed a novel human-like motion generation algorithm that analyzes human motion through functional principal component analysis features, enabling efficient synthesis of complex movements such as free motion and obstacle avoidance in free space. Building upon the concept of human motion analysis, Zhao et al. [148] applied these principles to rehabilitation by designing a tele-rehabilitation system that integrates natural human responses with big data analytics. Their system includes an upper limb rehabilitation robot that combines flexible ropes and exoskeleton components and is intended to assist clinicians in optimizing individualized rehabilitation programs. To further enhance rehabilitation management, they proposed a multidimensional training and assessment database to support a multilevel linked rehabilitation system. Extending robotic applications to lower extremity rehabilitation, Miller-Jackson et al. [149] designed a soft pneumatic actuator-driven exoskeleton for hip flexor rehabilitation, providing more options for individuals with lower extremity mobility issues. Compared with not wearing the device, subjects experienced a 43.5% reduction in muscle signals when lifting their legs while wearing the device, suggesting that the device is effective in assisting with hip flexion and that a pneumatic rotary actuator-driven exoskeleton is a viable solution. In conjunction with these hardware-based solutions, digital platforms such as smartphone-based applications are also playing an increasingly important role in rehabilitation. Rossi et al. [150] investigated the use of a smartphone-based care management platform, myMobility, in the context of TKA rehabilitation.

### Personalized treatment planning

Precision medicine aims to tailor treatment strategies by classifying patients into subgroups based on variations in prognosis or treatment response, thereby optimizing therapeutic benefits while minimizing unnecessary interventions [151]. AI algorithms are well equipped to handle high-dimensional data, support predictive modeling, and inform personalized management strategies in clinical settings [152]. AI is transforming surgical planning and clinical decision-making by analyzing patient-specific data to predict outcomes, optimize procedures, and guide personalized strategies (Table 4) [32–36, 49, 153–160].

**Table 4:** Application of AI in personalized treatment planning

Algorithm	Application	References
ML model	Predicting functional outcome and quality of life after instrumental correction of adult spinal deformity	[32]
Cluster hierarchy	Predicting the outcome of postoperative patient self-assessment and providing an accurate risk–benefit analysis for various combinations of surgical techniques	[153]
CART model and cluster analysis	Predicting the risk of postoperative complications and unplanned readmission in surgically treated adult spinal deformity cases	[157]
Factorial analysis	Predicting the establishment of a specific gait model for THA patients	[155]
ML model	Predicting opioid use after THA	[33]
ML model	Predicting the ASES score after ARCR with relatively high accuracy	[36]
ML model	Predicting short-term outcomes after ORIF treatment of ankle fractures	[35]
ML model	Predicting the recurrence of PJI after TKA	[34]
Random forest ML analytical model	Predicting outcomes after irrigation and debridement for PJI	[158]
CNN	Analyzing movement during activities like walking, running, and sidestepping to detect ACL risks	[156]
ML model	Measuring the ACL injury risks	[159]
ML model	Predicting primary ACL injuries	[160]
DL model	Predicting the need for TKA based on knee radiographs	[49]
Classification tree algorithm	Assessing the risk of cervical spine injury in pediatric patients	[154]

*ML* machine learning, *CART* classification and regression tree,* THA* total hip arthroplasty, *ASES* American shoulder and elbow surgeons, *ARCR* arthroscopic rotator cuff repair, *ORIF* open reduction internal fixation, *PJI* periprosthetic joint infection, *TKA* total knee arthroplasty, *CNN* convolutional neural network, *ACL* anterior cruciate ligament, *DL* deep learning

Predictive analytical models have been applied extensively in adult spinal deformity surgeries. For example, Ames et al. [32] used an ML model to predict functional outcome and quality of life after instrumental correction of adult spinal deformity. This model takes multiple factors into account, tailoring predictions to the unique circumstances of each patient, thereby improving preoperative counseling and decision-making. Building on this work, the same team later introduced a refined classification framework based on hierarchical clustering [153], which enhances decision-making by identifying data patterns. This system offers 2-year risk‒benefit grids, guiding surgeons in selecting the most appropriate strategies, such as posterior fusion, intervertebral implantation, or pedicle subtraction osteotomy.

Model interpretability plays a crucial role in clinical adoption. Transparent models allow clinicians to understand key predictive factors, such as age, comorbidities, and surgical complexity, ensuring alignment with clinical reasoning. This fosters trust between patients and providers and mitigates potential biases, improving the generalizability of models across diverse populations. Interpretability also enhances personalized treatment planning, as demonstrated by Pellisé et al. [157]. The classification and regression tree algorithm was used to predict complications such as nonunion, vertebral kyphosis, and pedicle screw loosening, providing preoperative insights into the risk of implant failure and guiding individualized interventions. Similarly, Cattaneo et al. [155] applied factorial analysis to study gait changes post-THA, demonstrating how different surgical approaches, i.e., posterior vs. anterior, impact recovery trajectories. These findings emphasize that AI-powered predictive models align surgical strategies with individual patient needs, enhancing postoperative outcomes.

AI is likewise used for postsurgical evaluation and prediction of complications after other procedures, such as THA, arthroscopic rotator cuff repair (ARCR), ankle fracture open reduction internal fixation (ORIF), TKA, and flushing and debridement after periprosthetic joint infection (PJI). To predict opioid use after THA, Karhade et al. [33] designed a preoperative ML algorithm that analyzed a total of 5507 patients. The study revealed that 345 patients had long-term postoperative opioid use and identified predictors of long-term opioid prescription. The best model had an AUC of 0.77, indicating high net returns. Potty et al. [36] proposed a new ML algorithm to predict the American Shoulder and Elbow Surgeons score after ARCR with relatively high accuracy. Merrill et al. [35] used ML to predict short-term outcomes after ORIF for ankle fractures. ML accurately predicted several comorbidities associated with poor short-term outcomes after ORIF for ankle fractures. Klemt et al. [34] developed and validated 3 ML models to predict the recurrence of PJI after TKA. The factors related to recurrence after TKA were irrigation and debridement, > 4 previous open surgeries, metastatic disease, drug abuse, acquired immune deficiency syndrome, enterococcal infection, and obesity. All the ML models achieved good discrimination performance. Shohat et al. [158] designed a retrospective study that collected data on 1174 THA and TKA procedures. They used a randomized forest ML model in assessing 52 variables to predict outcomes after irrigation and debridement for PJI. The model achieved good discrimination (AUC = 0.74) and high accuracy. In addition, the model identified 10 significant factors associated with failure, such as positive blood cultures and elevated C-reactive protein levels.

AI has gradually transformed sports medicine, especially in the area of ACL injury risk prediction. AI methods have facilitated the identification of biomechanical factors associated with ACL injury risk, supporting subsequent developments in imaging-based and sensor-driven predictive models [156, 159–161]. Pedoia et al. [161] used 3D MRI-based statistical shape modeling to uncover anatomical features associated with ACL injury, such as specific intercondylar width and posterior tibial slope values. Johnson et al. [156] developed a DL-based system that uses a pretrained CNN to monitor knee joint movements in real time. Movement during activities such as walking, running, and sidestepping is analyzed to detect ACL injury risk, offering valuable insights for athletic training and injury prevention. Taborri et al. [159] quantified ACL injury risk by assessing stability and load absorption through inertial sensors and optoelectronic devices. Research has demonstrated that the landing error score system strongly correlates with the risk of ACL injury. Tamimi et al. [160] built a supervised ML-based predictive model using knee morphology data from MRI scans to predict primary ACL injury, achieving 92% testing accuracy and highlighting the role of AI in structural analysis and early intervention.

AI has also demonstrated value in predicting the progression of chronic diseases. In a recent study, Leung et al. [49] developed a DL prediction model for OA progression risk that could directly predict the need for TKA based on knee radiographs. Compared with the binary outcome model using a standard grading system, the proposed DL model better predicted the risk of TKA for OA. Bertsimas et al. [154] demonstrated the value of ML in improving clinical decision-making in pediatric trauma patients. They retrospectively assessed the risk of cervical spine injury in pediatric patients in conjunction with an optimal classification tree algorithm, achieving a sensitivity of 93.3% and a specificity of 82.3%. These models enable health care professionals to make informed, data-driven decisions, ensuring timely interventions and optimal patient outcomes.

By facilitating the early detection of potential risk factors, interpretable computational models contribute to the development of individualized preventive strategies and may assist in improving clinical decision-making. Within orthopedic practice, such models can aid in tailoring treatment plans, refining preoperative assessments, and supporting postoperative management. In areas such as complication risk assessment, rehabilitation planning, and injury prevention, data-driven approaches offer valuable support for predictive analyses. The capacity to process complex clinical data, recognize subtle associations, and provide interpretable outputs underscores the potential utility of these tools in the context of orthopedics and sports medicine.

## Limitations and future perspectives

Progress in AI applications for musculoskeletal imaging and orthopedic care has been substantial; however, numerous limitations persist that affect the reliability and integration of AI-based methods into clinical practice.

### Limitations in data and model design

The training of AI models heavily depends on large-scale, high-quality, and diverse datasets. However, in orthopedics, data scarcity remains a major issue [162]. Most current studies have relied on imaging data from single centers, specific equipment, and defined populations, which limits the representativeness and generalizability of the results [40, 56, 163, 164]. Additionally, the annotation process involves subjectivity and lacks uniform standards [41, 56]; for example, the use of radiology reports or manual segmentation as reference standards introduces potential bias into the model.

The inherent “black box” nature of AI systems further increases uncertainty in clinical applications [165]. Although deep models can achieve high predictive accuracy, they usually lack interpretability and fail to provide clinicians with understandable decision paths, especially in complex clinical scenarios. Although post hoc visualization methods can assist with partial interpretation, they do not fully replace traceable clinical logic [162]. Furthermore, AI models are vulnerable to adversarial perturbations [166], where small modifications to input images may result in incorrect predictions, posing risks to model safety and reliability.

### Technical and operational challenges in clinical application

The clinical application of AI is currently limited by various technical and operational challenges. First, most AI systems are designed for specific tasks such as image classification or structure recognition, and are unable to address broader clinical workflows, including disease correlation and individualized treatment planning [66, 85]. Moreover, AI performs poorly in subjective assessments and cannot handle ambiguous or uncertain information [92], which further limits its utility in clinical scenarios. Second, AI systems are highly dependent on data and computational resources. In practice, many models require significant computing power and specific software-hardware configurations [93, 162], making deployment in resource-limited settings difficult. Model performance is also affected by image quality and the presence of anatomical landmarks, and variations in imaging protocols between hospitals add further complexity to deployment [93].

From a translational perspective, clinical validation remains insufficient. Many studies are based on data from single institutions, and model generalizability across different regions, equipment, and populations has not been adequately tested. For example, only 11% of fracture detection studies reported external geographical validation [167], significantly limiting the clinical applicability of the results. Adaptability to specific populations and complex cases also remains a critical concern. Current AI systems have yet to receive FDA approval for pediatric fracture detection [85], as pediatric fractures often exhibit unique radiographic features that necessitate specialized detection algorithms [168]. Additionally, the performance of AI models in detecting stress fractures, pathological fractures, and abnormalities in bone structure still requires improvement [121]. Beyond these challenges, the lack of long-term follow-up data and limited validation of real-world clinical outcomes hinder the translation of AI-based methods from research settings to routine orthopedic practice. Short-term diagnostic accuracy does not necessarily equate to long-term therapeutic benefits, and few studies have established definitive links between AI-assisted interventions and patient prognoses over time [41, 125].

Therefore, to achieve effective translation and clinical adoption of AI-based methods in orthopedics, it is necessary to optimize deployment processes; enhance model functionality, strengthen multicenter, multimodal, large-scale clinical validation; and promote real-world research design to improve adaptability and reliability in complex clinical environments.

### Acceptance, cost, and ethical considerations

AI still faces considerable barriers to clinical acceptance. One major issue is the lack of clinician trust in “black box” decision-making systems, especially in critical diagnostic scenarios where the reasoning path is not transparent [169, 170]. Additionally, health care providers, particularly less experienced interns or junior doctors, may become overly reliant on AI systems, which could impair the development of their independent clinical judgment [66].

Another significant challenge involves ethical and legal regulation [171]. At present, there is no unified standard for obtaining informed consent for the use of data in model training, and issues such as algorithmic fairness, justice, and data privacy lack systematic evaluation. As developers are not medical professionals, their legal responsibility remains undefined, and clearer regulatory guidance and stakeholder negotiation are urgently needed. Furthermore, AI models often inherit biases from training data, which may amplify systemic inequities related to race or gender. To date, most studies have not considered population diversity or ethnic differences [172].

### Future perspectives

In summary, although AI methods are developing rapidly and extensively in orthopedics, their clinical application is still limited by several factors, including data quality and scale, model stability, the complexity of clinical environments, medical trust, and regulatory mechanisms. To advance the field, future research should focus on the following aspects: establishing high-quality, multicenter, ethnically diverse data platforms to promote standardized data sharing; developing more interpretable model architectures to increase clinical trust; expanding AI capabilities to cover the full diagnostic and treatment chain, including disease progression prediction, surgical planning, and long-term outcome evaluation; strengthening ethical review and regulatory frameworks to clarify the responsibilities of developers and users; and promoting long-term, multiregional, real-world clinical validation and iterative optimization. Only by addressing these challenges can AI in orthopedics truly evolve from a “laboratory technology” to a “clinical productivity tool”.

## Conclusions

**Fig. 3:**
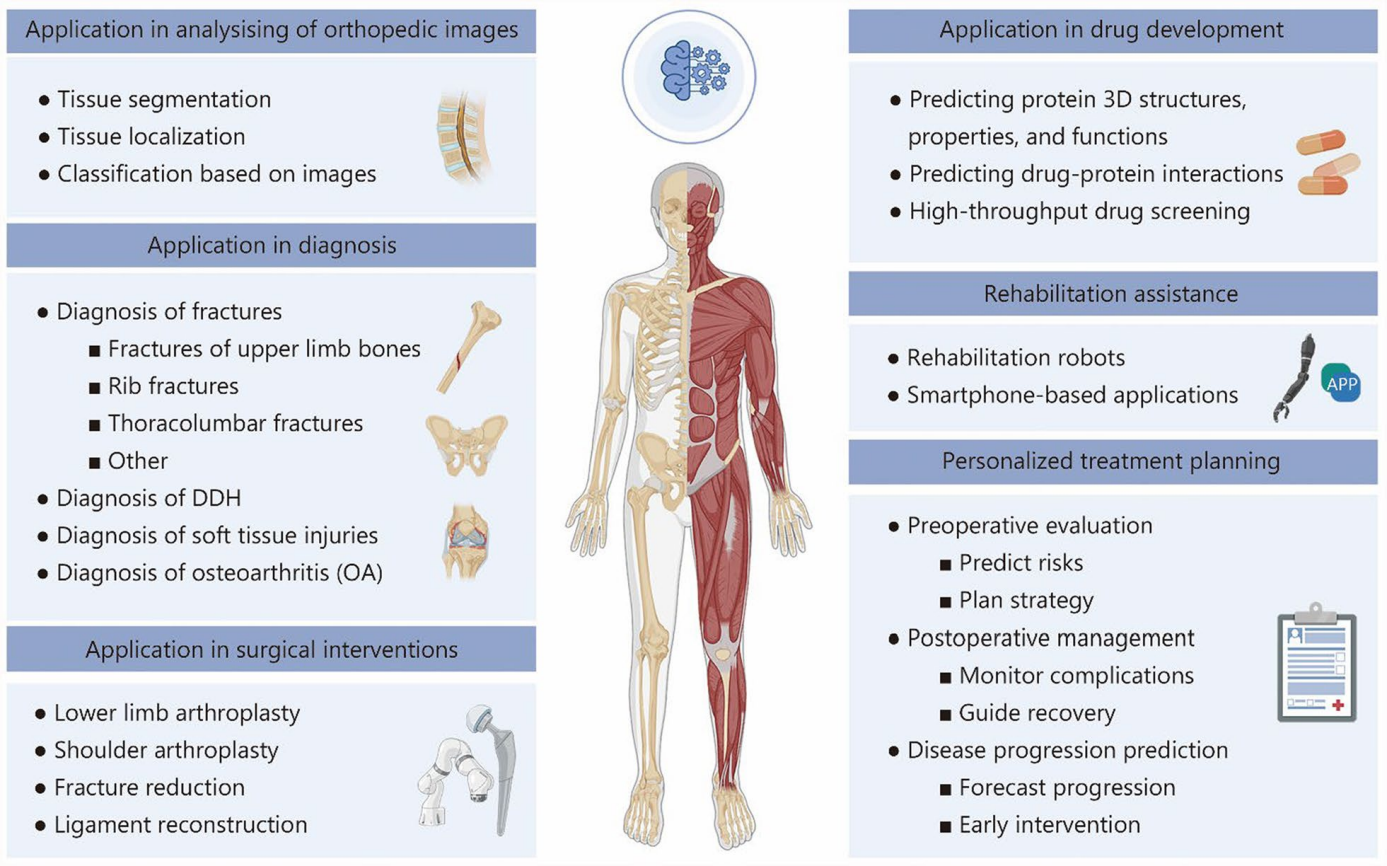
Comprehensive applications of AI in orthopedics. DDH developmental dysplasia of the hip, 3D three-dimensional

This review provides a comprehensive overview of the current applications of computational technologies in orthopedics (Fig. 3), with a particular focus on their roles in imaging analysis, clinical diagnosis, and various aspects of treatment, including surgical planning, drug development, rehabilitation, and personalized care. These tools have been employed to enhance the precision and efficiency of image interpretation, support clinical decision-making, and contribute to individualized therapeutic strategies. At the same time, this review also identifies a range of limitations that continue to hinder their broader implementation. These include constraints related to data availability and model generalizability, challenges in integrating such tools into complex clinical workflows, and concerns surrounding ethical oversight, regulatory standards, and long-term clinical validation. Future research should prioritize the development of interpretable and robust systems, the construction of diverse and high-quality datasets, and the establishment of multidisciplinary frameworks to ensure the responsible and effective incorporation of these technologies into orthopedic practice.

## Data Availability

Not applicable.

## Abbreviations

2D: Two-dimensional
3D: Three-dimensional
ACL: Anterior cruciate ligament
AI: Artificial intelligence
ARCR: Arthroscopic rotator cuff repair
AUC: Area under the curve
CASP: Critical assessment of protein structure prediction
CB513: CullPDB 513
CBCT: Cone-beam computed tomography
CNN: Convolutional neural networks
CT: Computed tomography
DCNN: Deep convolutional neural network
DDH: Developmental dysplasia of the hip
DeepFRI: Deep functional residue identification
DL: Deep learning
DNN: Deep neural networks
HOG: Histogram of oriented gradient
IHDI grade: International Hip Dysplasia Institute grade
ML: Machine learning
MR: Magnetic resonance
MRI: Magnetic resonance imaging
MURA: Musculoskeletal radiograph
OA: Osteoarthritis
ORIF: Open reduction internal fixation
PHOG: Pyramidal histogram of oriented gradient
PJI: Periprosthetic joint infection
R&D: Research and development
SD: Standard deviation
SVM: Support vector machine
THA: Total hip arthroplasty
TKA: Total knee arthroplasty
